# Supplementary material for: Study on Lavender Essential Oil Chemical Compositions by GC-MS and Improved pGC
Source: Molecules. 2020 Jul 10;25(14):3166. doi: 10.3390/molecules25143166 (PMC7397202; doi:10.3390/molecules25143166)

Supplementary Material for:

Study on lavender essential oil chemical compositions by GC-MS~~,~~ and improved pGC~~ and autodock~~~~,~~

Formatted: Font: (Asian) SimSun, 16 pt, Bold

Figure S1. GC-MS equipped with HP-5 ms, HP-1 and HP-INNOWax capillary columns TiC spectra for Lavender essential oil.

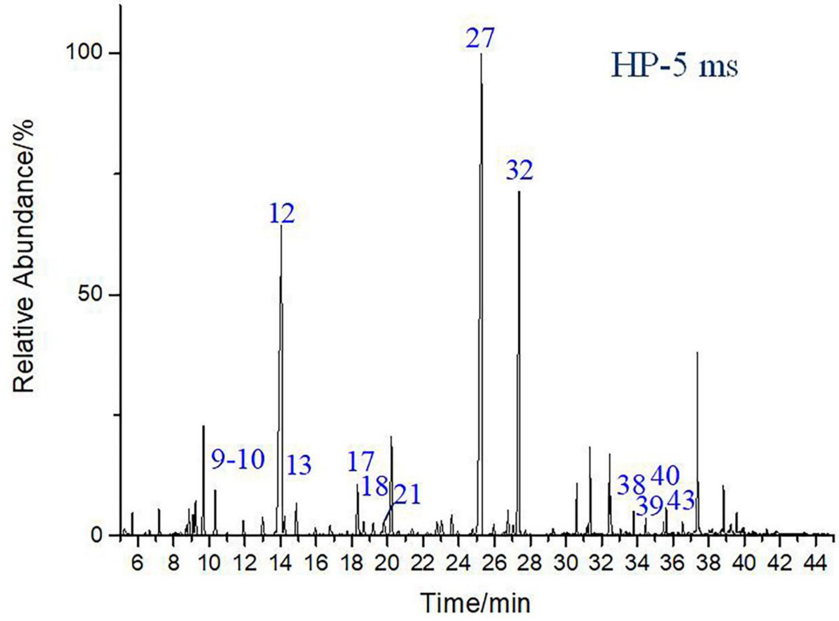

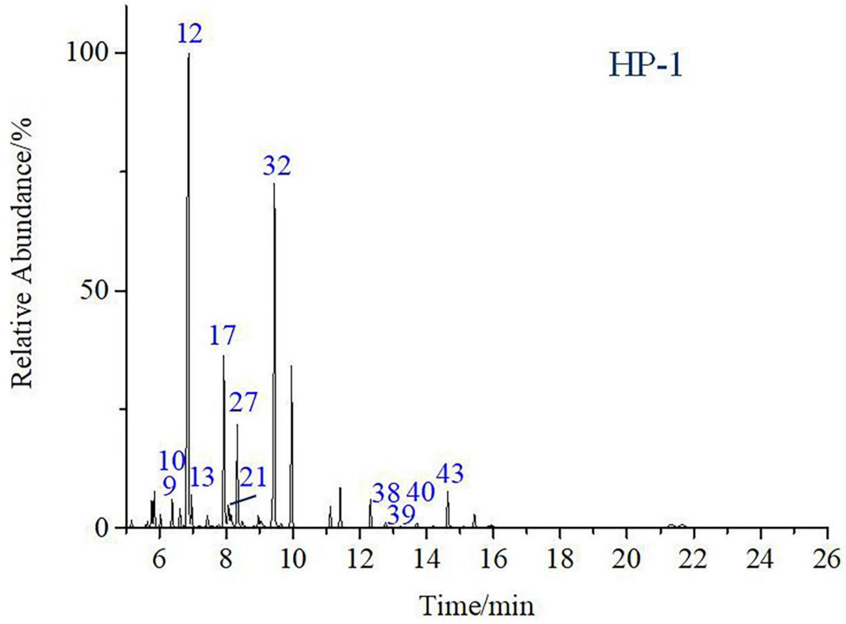
HP-1
12
32
17
27
10
9
13
21
38
39
40
43
Relative Abundance/%
100
50
0
6
8
10
12
14
16
18
20
22
24
26
Time/min

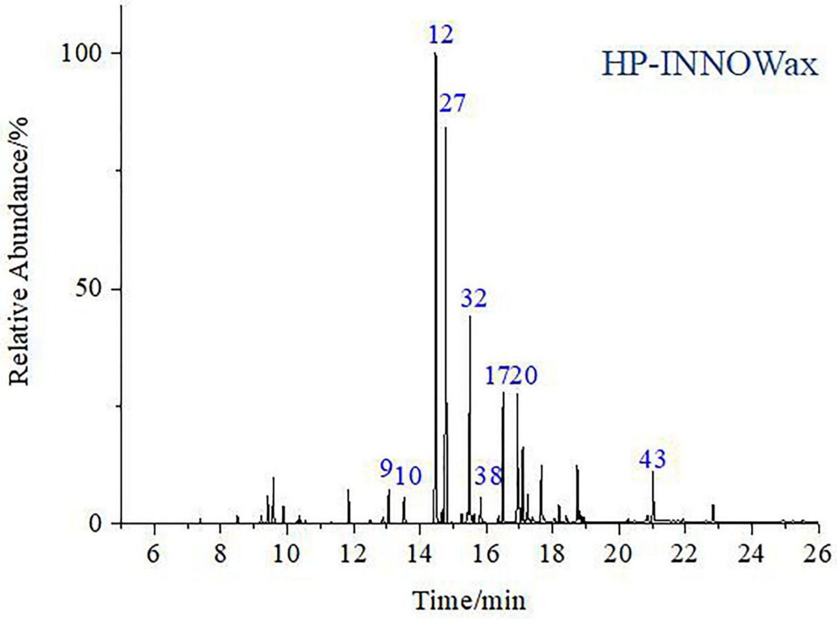
HP-INNOWax
12
27
32
17
20
9
10
38
43
Relative Abundance/%
100
50
0
6
8
10
12
14
16
18
20
22
24
26
Time/min

Figure S2. GC-EI-QTOF-MS Tic spectra for compounds 9, 10, 12, 13, 17, 18, 19, 21, 27, 29, 32, 33, 38, 39, 40, 41, 43, 45, 46, 47.

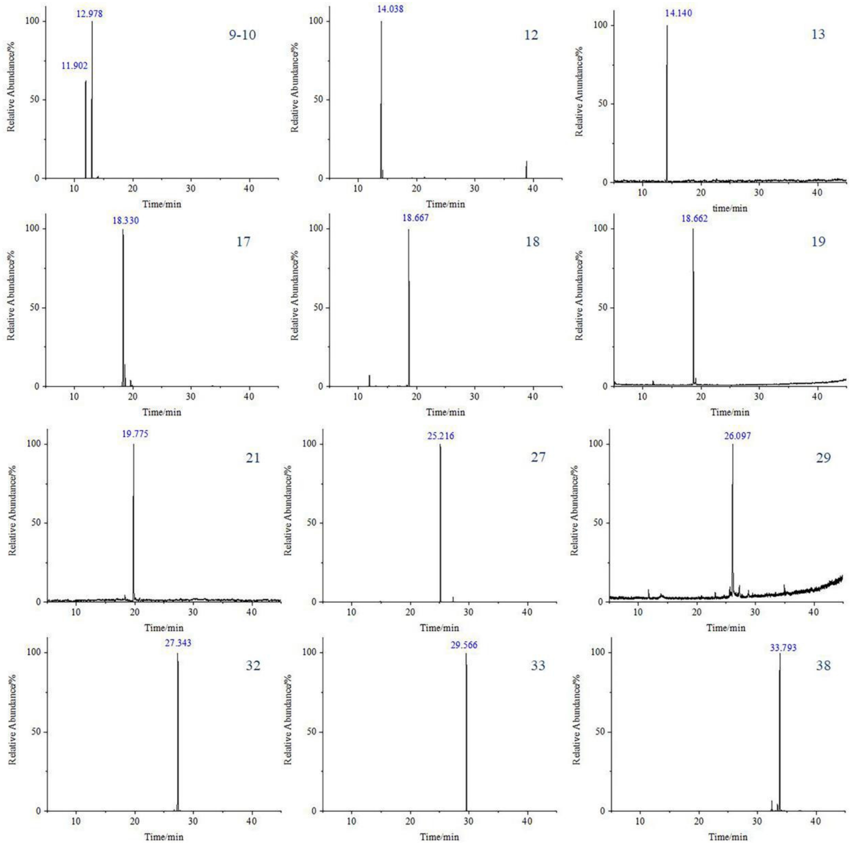

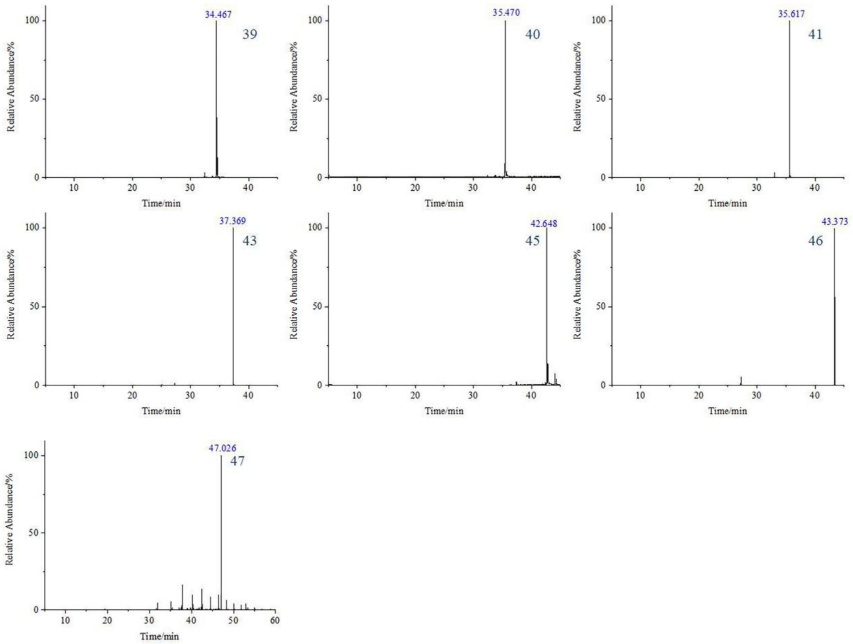
34.467
39
35.470
40
35.617
41
37.369
43
42.648
45
43.373
46
47.026
47
Relative Abundance/%
Time/min

Figure S3. GC-EI-QTOF-MS spectra for compounds 9, 10, 12, 13, 17, 18, 19, 21, 27, 29, 32, 33, 38, 39, 40, 41, 43, 45, 46, 47.

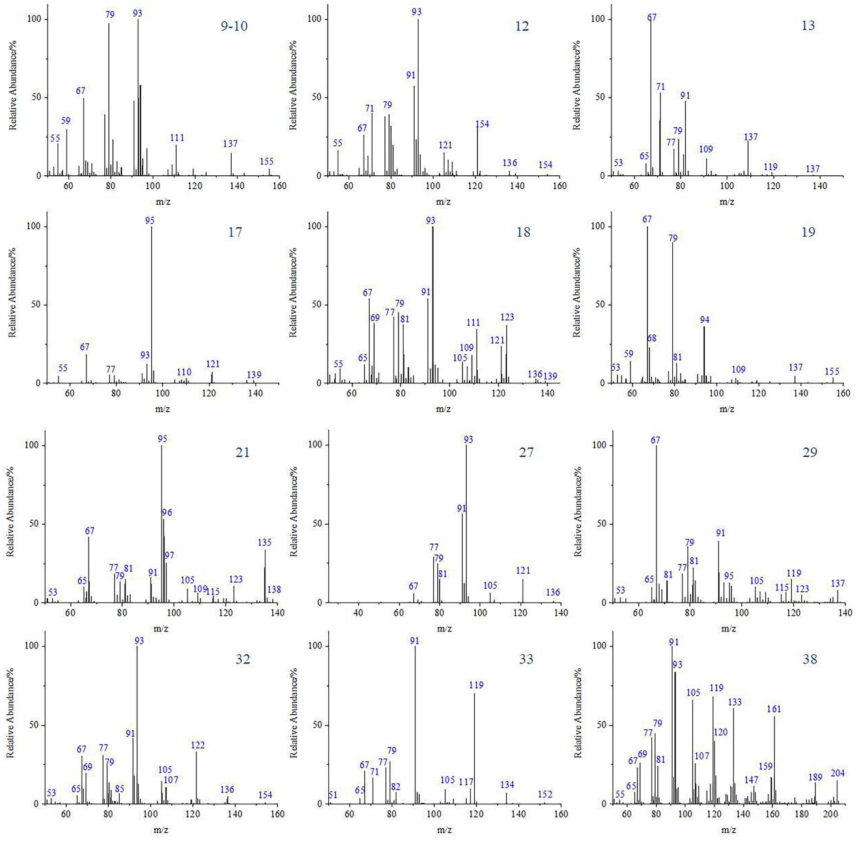

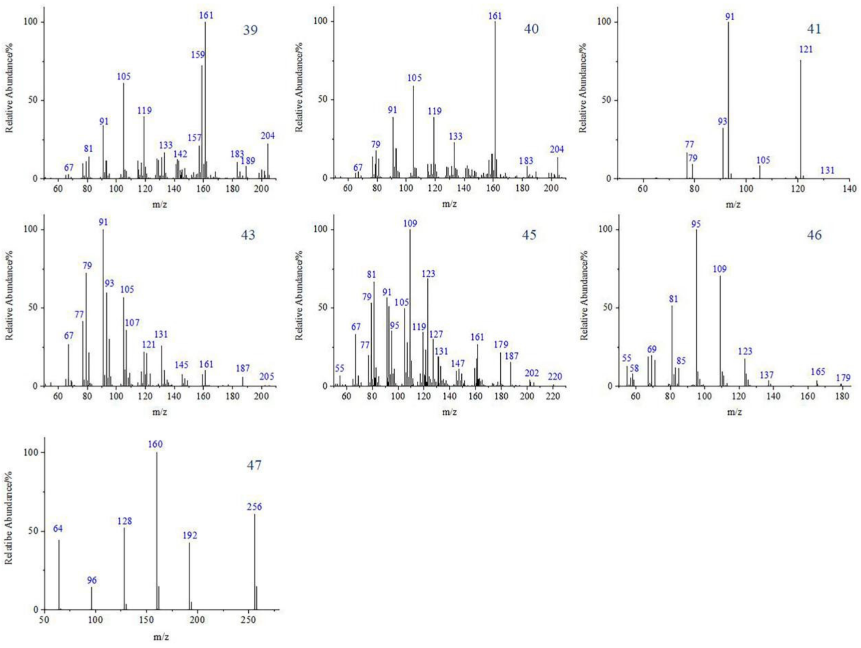
39
161
159
105
119
91
81
67
133
142
157
183
189
204
40
161
105
119
91
79
67
133
183
204
41
91
121
93
77
79
105
131
43
91
79
93
105
77
67
107
131
121
145
161
187
205
45
109
81
123
79
91
105
67
95
119
127
77
131
147
161
179
187
55
202
220
46
95
109
81
69
55
58
85
123
137
165
179
47
160
256
128
64
192
96
Relative Abundance/%
m/z

Spectral data

Cis-Linalool oxide and trans-Linalool oxide (9-10). $C_{10}H_{18}O_2$; RI (HP-5 ms) 1069; EI-MS 70 eV, m/z (rel. int.): 155(4, $M^{+\cdot}$), 137(14, $[M-H_2O]^{+\cdot}$), 125(3), 119(5), 111(20), 109(7), 106(4), 97(17), 95(11), 94(58), 93(100), 91(48), 81(24), 79(97), 77(39), 67(50), 59(30), 55(21), 53(5). ^{13}C NMR ($CDCl_3$, 600 MHz) δ 24.18, 24.33 (8C), 26.02, 26.83 (1C), 26.49 (4C), 27.25, 27.44 (7C), 37.48, 37.93 (3C), 71.12, 71.22 (6C), 82.79, 83.05 (2C), 85.54 (5C), 111.32, 111.58 (10C), 143.71, 144.32 (9C).

Linalool (12). $C_{10}H_{18}O$; RI (HP-5 ms) 1102; EI-MS 70 eV, m/z (rel. int.): 154(1, $M^{+\cdot}$), 139(2, $[M-CH_3]^{+\cdot}$), 136(3), 121(32), 107(10), 105(15), 96(5), 94(14), 93(100), 92(23), 91(57), 83(5), 82(3), 81(20), 80(32), 79(39), 78(3), 77(38), 72(3), 71(40), 69(13), 67(26), 65(5), 55(16). ^{13}C NMR ($CDCl_3$, 600 MHz) δ 17.67 (8C), 22.78 (10C), 25.67 (9C), 27.85 (5C), 42.04 (4C), 73.44 (3C), 111.64 (1C), 124.29 (6C), 131.91 (7C), 145.02 (2C).

Hotrienol (13). $C_{10}H_{16}O$; RI (HP-5 ms) 1105; EI-MS 70 eV, m/z (rel. int.): 137(1, $[M-CH_3]^{+\cdot}$), 119(2), 109(22), 106(2), 105(2), 93(3), 91(11), 83(3), 82(48), 81(14), 80(3), 79(24), 77(17), 71(53), 67(100), 65(8), 53(3). ^{13}C NMR ($CDCl_3$, 600 MHz) δ 18.66 (10C), 27.54 (9C), 45.77 (4C), 72.81 (3C), 112.00 (1C), 115.62 (8C), 124.85 (5C), 137.06 (6C), 139.62 (7C), 144.75 (2C).

(-)-Borneol (17). $C_{10}H_{18}O$; RI (HP-5 ms) 1161; EI-MS 70 eV, m/z (rel. int.): 139(2, $[M-CH_3]^{+\cdot}$), 136(2), 121(7), 110(3), 96(8), 95(100), 93(12), 91(5), 81(2), 79(5), 77(6), 67(18), 55(5). ^{13}C NMR ($CDCl_3$, 600 MHz) δ 13.31 (10C), 18.67 (8C), 20.18 (9C), 25.90 (6C), 28.27 (5C), 39.03 (3C), 45.09 (4C), 48.02 (7C), 49.96 (1C), 77.39 (2C).

Lavandulol (18). $C_{10}H_{18}O$; RI (HP-5 ms) 1166; EI-MS 70 eV, m/z (rel. int.): 139(1, $[M-CH_3]^{+\cdot}$), 136(2), 135(3), 123(37), 121(24), 111(34), 109(18), 107(10), 105(14), 95(9), 94(12), 93(100), 91(54), 83(11), 81(37), 79(45), 77(42), 71(7), 69(38), 68(11), 67(55), 65(12), 55(10), 53(7), 51(6). ^{13}C NMR ($CDCl_3$, 400 MHz) δ 17.83 (10C), 19.50 (9C), 25.74 (6C), 28.38 (3C), 49.96 (2C), 63.63 (1C), 113.14 (8C), 122.00 (4C), 132.79 (5C), 145.40 (7C).

2,2,6-trimethyl-6-vinyltetrahydro-2H-pyran-3-ol (19). $C_{10}H_{18}O_2$; RI (HP-5 ms) 1166; EI-MS 70 eV, m/z (rel. int.): 155(5, $[M-CH_3]^{+\cdot}$), 137(5), 109(4), 107(3), 97(4), 95(5), 94(36), 93(5), 91(6), 83(6), 81(13), 79(90), 77(7), 68(23), 67(100), 59(15), 55(5), 53(5). ^{13}C NMR ($CDCl_3$, 600 MHz) δ 24.23 (4C), 26.44 (11C), 27.17 (3C), 27.52 (10C), 30.94 (9C), 71.25 (5C), 73.58 (6C), 75.24 (2C), 110.52 (8C), 146.79 (7C).

Cryptone (21). $C_9H_{14}O$; RI (HP-5 ms) 1182; EI-MS 70 eV, m/z (rel. int.): 138(2, $M^{+\cdot}$), 136(4), 135(34), 123(12, $[M-CH_3]^{+\cdot}$), 121(24), 120(3), 119(3), 117(3), 115(5), 110(3), 109(6), 105(9), 97(26), 96(53), 95(100), 91(16), 83(6), 82(5), 81(16), 79(13), 77(19), 67(41), 66(7), 65(10), 53(3), 51(3). ^{13}C NMR ($CDCl_3$, 600 MHz) δ 19.47 (9C), 19.63 (8C), 25.25 (5C), 31.51 (7C), 37.42 (6C), 42.52 (4C), 129.69 (2C), 154.32 (3C), 200.10 (1C).

Linalyl acetate (27). $C_{12}H_{20}O_2$; RI (HP-5 ms) 1263; EI-MS 70 eV, m/z (rel. int.): 136(1), 121(15),

107(2), 105(6), 94(4), 93(100), 92(12), 91(56), 80(15), 79(25), 77(29), 69(2), 67(6), 65(1). ^{13}C NMR ($CDCl_3$, 400 MHz) δ 17.54 (12C), 22.16 (10C), 22.29 (11C), 23.54 (5C), 25.65 (8C), 39.65 (4C), 82.83 (3C), 113.04 (1C), 123.74 (6C), 131.76 (7C), 141.77 (2C), 169.94 (9C).

3,7-dimethylocta-1,7-diene-3,6-diol (29). $C_{10}H_{18}O$; RI (HP-5 ms) 1276; EI-MS 70 eV, m/z (rel. int.): 137(8, $[M-H_2O+H]^{+\cdot}$), 135(4), 134(3), 123(5), 119(15), 117(7), 115(6), 109(7), 107(8), 105(10), 96(11), 95(13), 93(13), 91(40), 82(14), 81(22), 79(37), 77(18), 71(14), 69(9), 68(12), 67(100), 65(10), 55(3), 53(3), 51(3). ^{13}C NMR ($CDCl_3$, 600 MHz) δ 17.76, 17.89 (10C), 28.16, 28.28 (9C), 29.16, 29.36 (5C), 37.72, 38.23 (4C), 72.93 (3C), 75.76, 76.22 (6C), 110.94, 110.98 (8C), 111.88, 112.00 (1C), 144.94 (2C), 147.49 (7C).

Lavandulol acetate (32). $C_{12}H_{20}O_2$; RI (HP-5 ms) 1295; EI-MS 70 eV, m/z (rel. int.): 154(1, $[M-COCH_3+H]^{+\cdot}$), 136(5), 123(3), 122(33), 107(11), 105(15), 94(13), 93(100), 92(17), 91(42), 85(7), 81(9), 80(14), 79(24), 77(31), 69(19),6 8(9), 67(30), 65(6), 55(1), 53(4), 51(3). ^{13}C NMR ($CDCl_3$, 400 MHz) δ 17.80 (9C), 19.90 (8C), 20.97 (12C), 25.75 (7C), 28.54 (4C), 46.02 (3C), 65.83 (10C), 112.36 (1C), 121.54 (5C), 132.93 (6C), 144.84 (2C), 171.11 (11C).

(E)-7-hydroxy-3,7-dimethylocta-1,5-dien-3-ylacetate (33). $C_{12}H_{20}O_3$; RI (HP-5 ms) 1344; EI-MS 70 eV, m/z (rel. int.): 152(1), 134(7), 120(1), 119(70), 117(10), 115(4), 109(3), 105(9), 103(2), 93(6), 92(8), 91(100), 82(8), 81(2), 79(27), 77(23), 71(16), 67(21), 65(4), 51(1). ^{13}C NMR ($CDCl_3$, 600 MHz) δ 22.15 (12C), 23.65 (9C), 29.88 (10C), 42.05 (4C), 70.73 (7C), 82.35 (3C), 113.50 (1C), 120.96 (5C), 141.49 (6C), 142.13 (2C), 169.89 (11C).

(E)-β-Fanesene (38). $C_{15}H_{24}$; RI (HP-5 ms) 1459; EI-MS 70 eV, m/z (rel. int.): 204(15, $M^{+\cdot}$), 189(14, $[M-CH_3]^{+\cdot}$), 175(4), 161(55), 159(17), 147(11), 133(61), 121(18), 120(40), 119(68), 117(14), 115(9), 109(12), 107(26), 105(66), 93(84), 91(100), 81(24), 79(45), 77(42), 69(26), 67(23), 65(7), 55(3), 51(3). ^{13}C NMR ($CDCl_3$, 400 MHz) δ 16.02 (14C), 17.68 (15C), 25.69 (12C), 26.61 (5C), 31.41 (4C), 39.70 (8C), 113.03 (1C), 115.70 (13C), 124.01 (6C), 124.34 (10C), 131.30 (11C), 135.38 (7C), 138.99 (2C), 146.12 (3C).

D-Germacrene (39). $C_{15}H_{24}$; RI (HP-5 ms) 1480; EI-MS 70 eV, m/z (rel. int.): 204(22, $M^{+\cdot}$), 202(5), 200(6), 198(4), 189(8, $[M-CH_3]^{+\cdot}$), 183(11), 161(100), 159(72), 157(21), 143(11), 142(12), 141(9), 133(17), 131(13), 129(12), 128(13), 119(39), 117(10), 115(12), 105(61), 93(11), 91(34), 81(14), 79(11), 77(10), 67(3), 65(3). ^{13}C NMR ($CDCl_3$, 600 MHz) δ 15.91 (14C)，19.33 (13C), 20.76 (12C), 26.51 (8C), 29.24 (2C), 32.75 (11C), 34.50 (3C), 40.71 (9C), 52.91 (7C), 109.02 (15C), 129.65 (1C), 133.55 (6C), 133.99 (10C),135.49 (5C), 148.88 (4C).

γ-Cadinene (40). $C_{15}H_{24}$; RI (HP-5 ms) 1514; EI-MS 70 eV, m/z (rel. int.): 204(13, $M^{+\cdot}$), 183(8), 162(12), 161(100), 159(15), 157(11), 133(22), 119(39), 105(59), 93(19), 91(39), 81(12), 79(17), 77(13), 67(4), 65(3). ^{13}C NMR ($CDCl_3$, 600 MHz) δ 15.12 (15C), 21.55 (12C), 23.90 (13C), 25.72 (8C), 26.21 (2C), 26.53 (9C), 30.49 (11C), 36.31 (3C), 44.19 (7C), 45.12 (1C), 46.90 (6C), 103.15 (14C), 122.43 (5C), 134.76 (4C), 153.39 (10C).

11-hydroxy-α-santal-9-ene (41). $C_{15}H_{24}O$; RI (HP-5 ms) 1519; EI-MS 70 eV, m/z (rel. int.):131(22), 122(2), 121(76), 119(1), 105(8), 94(3), 93(100), 91(33), 79(9), 77(17), 65(1). ^{13}C NMR ($CDCl_3$, 600 MHz) δ 10.69 (15C), 17.38 (14C), 19.50 (2C), 19.62 (4C), 26.95 (3C), 29.92 (12C), 29.94 (13C), 30.99 (5C), 31.23 (1C), 37.01 (8C), 38.42 (6C), 46.27 (7C), 70.75 (11C), 124.18 (9C), 139.43 (10C).

β-Caryophyllene oxide (43). $C_{15}H_{24}O$; RI (HP-5 ms) 1585; EI-MS 70 eV, m/z (rel. int.): 205(1, $[M-CH_3]^{+\cdot}$), 187(6), 161(10), 159(8), 145(8), 133(10), 131(26), 121(21), 119(21), 107(36), 105(57), 95(30), 93(60), 91(100), 81(21), 79(72), 77(42), 67(27), 55(3). ^{13}C NMR ($CDCl_3$, 600 MHz) δ 16.99 (12C), 21.62 (15C), 27.21 (2C), 29.80 (7C), 29.89 (6C), 30.19 (14C), 34.02 (11C), 39.16 (3C), 39.76 (10C), 48.73 (9C), 50.76 (1C), 59.82 (4C), 63.75 (5C), 112.74 (13C), 151.83 (8C).

(3S,6R,9R)-2-(hydroxymethyl)-5,5,9-trimethyltricyclo[7.2.0.0(3,6)]undecan-2-ol (45). $C_{15}H_{26}O_2$; RI (HP-5 ms) 1811; EI-MS 70 eV, m/z (rel. int.): 220(1, $[M-H_2O]^{+\cdot}$), 205(2), 202(5), 187(15), 179(21), 161(27), 159(11), 149(7), 147(11), 145(10), 133(13), 131(19), 127(30), 123(69), 121(23), 119(34), 109(100), 107(28), 105(49), 95(35), 93(51), 91(57), 81(67), 79(53), 77(20), 67(33), 55(7). ^{13}C NMR (CD_3COCD_3, 600 MHz) δ 20.69 (12C), 21.40 (2C), 27.64 (3C), 28.52 (13C), 30.95 (6C), 33.81 (14C), 34.59 (7C), 34.72 (11C), 35.68 (9C), 38.60 (10C), 39.14 (4C), 43.49 (1C), 43.62 (5C), 69.42, 70.65 (8C), 73.31 (15C).

(6R,10R)-6,10,14-Trimethyl-2-pentadecanone (46). $C_{18}H_{36}O$; RI (HP-5 ms) 1846; EI-MS 70 eV, m/z (rel. int.): 179(1), 165(4), 137(4), 125(4), 124(8), 123(18), 111(7), 110(9), 109(71), 97(4), 96(10), 95(100), 85(11), 83(11), 81(51), 71(16), 69(20), 67(19), 59(4), 58(8), 57(6), 55(13). ^{13}C NMR ($CDCl_3$, 600 MHz) δ 19.58 (16C), 19.74 (17C), 21.44 (4C), 22.62 (15C), 22.72 (18C), 24.42 (8C), 24.79 (12C), 27.98 (14C), 29.85 (1C), 32.67 (6C), 32.78 (10C), 36.49 (5C), 37.23 (9C), 37.28 (7C), 37.40 (11C), 39.36 (13C), 44.15 (3C), 209.38 (2C).

S_8 (47). S_8; RI (HP-5 ms) 2031; EI-MS 70 eV, m/z (rel. int.): 257(15, $[M+H]^{+\cdot}$), 256(60, $M^{+\cdot}$), 193(5), 192(43), 161(15), 160(100), 129(3), 128(52), 96(14), 64(45).

Figure S4. ^{13}C spectra for linalool oxide (9-10).

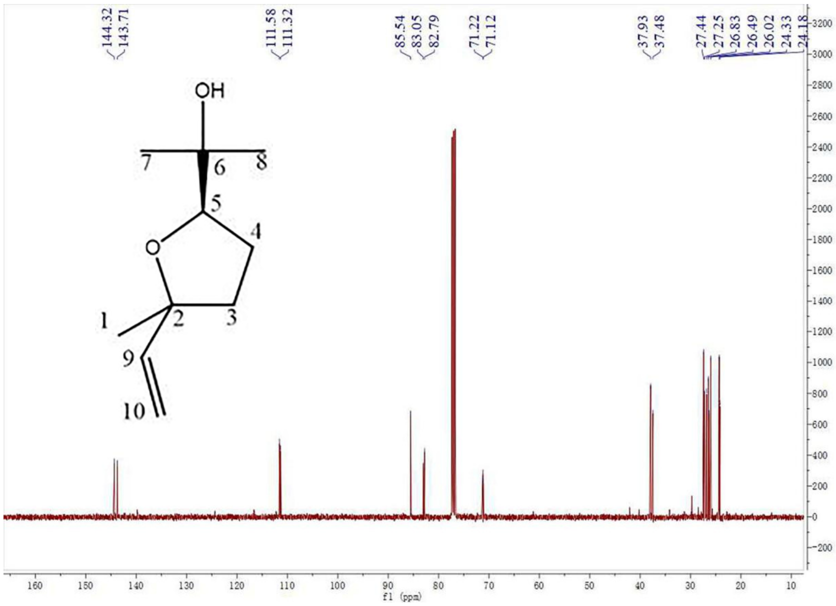

Figure S5. ^{13}C spectra for Linalool (12).

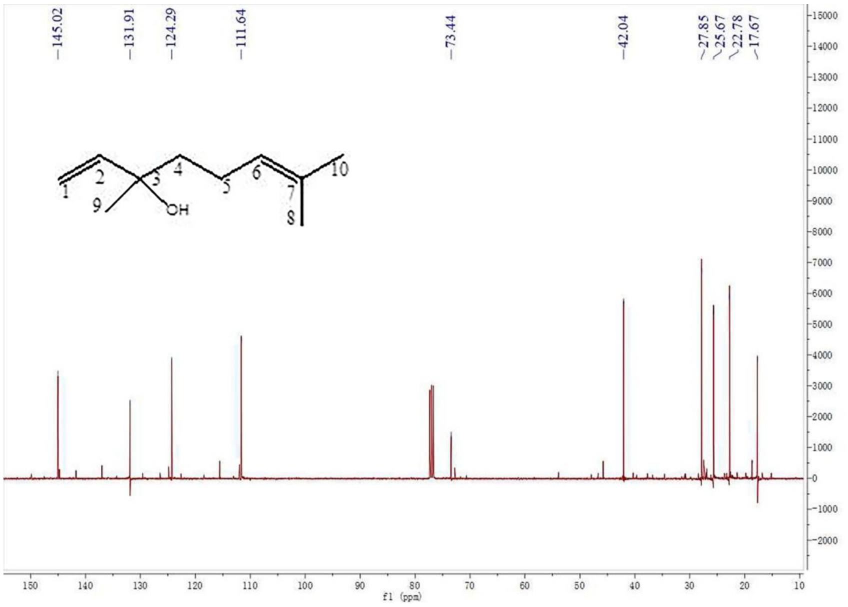

Figure S6. ^{13}C spectra for Hotrienol (13).

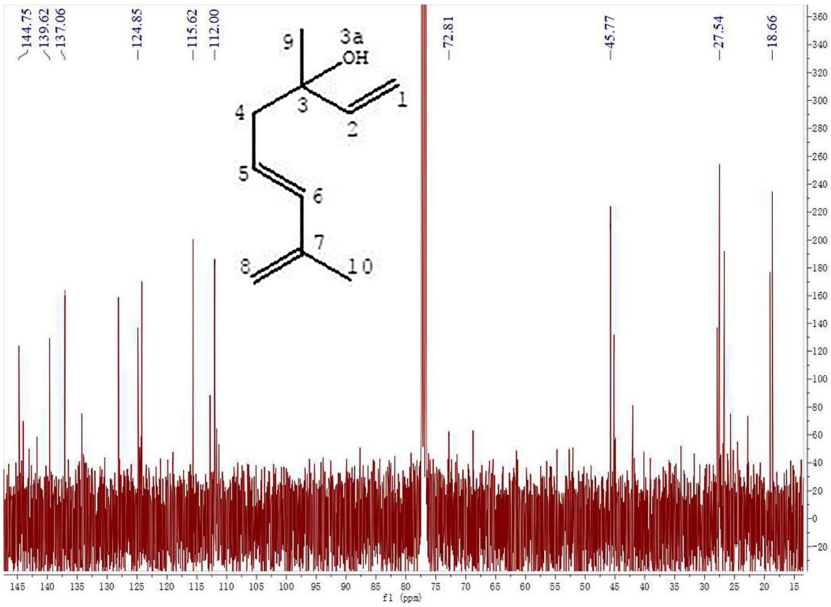

Figure S7. ^{13}C spectra for (-)-borneol (17).

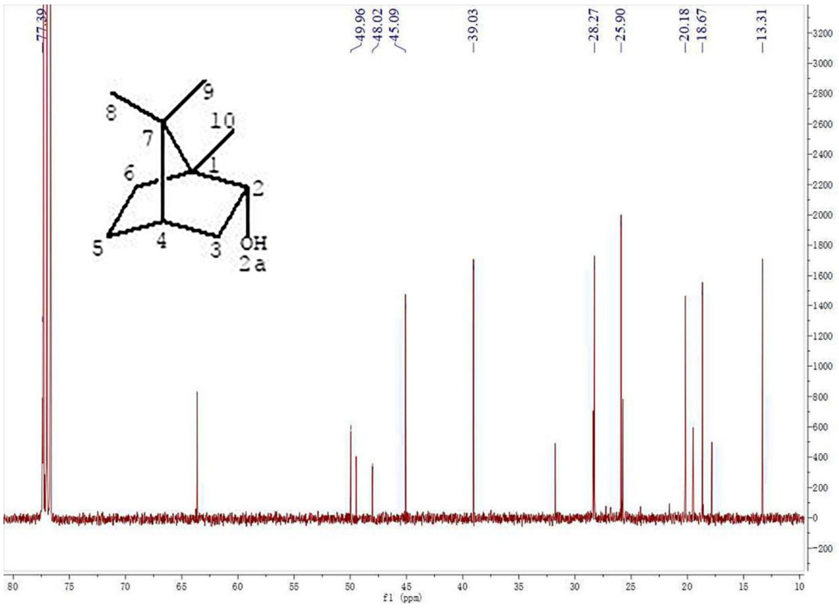

Figure S8. ^{13}C spectra for lavandulol (18).

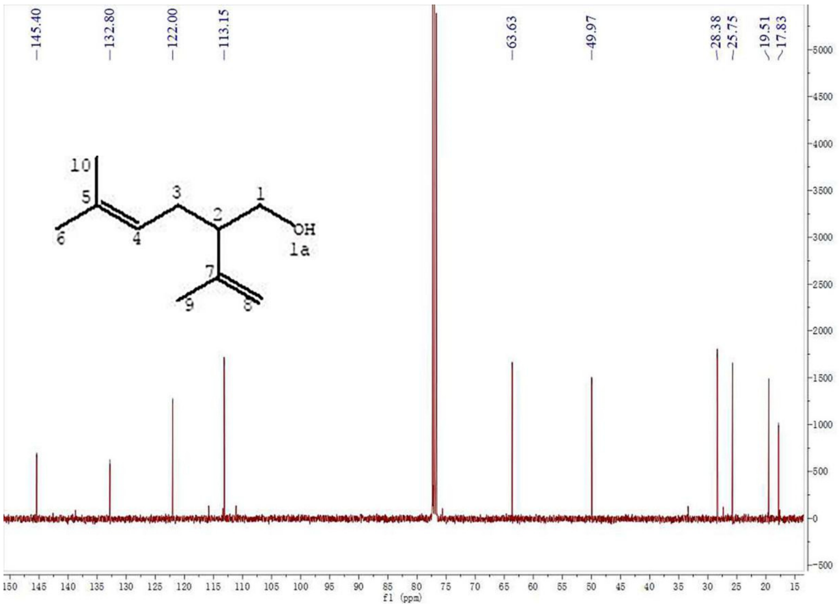

Figure S9. ^{13}C spectra for 2,2,6-trimethyl-6-vinyltetrahydro-2H-pyran-3-ol (19).

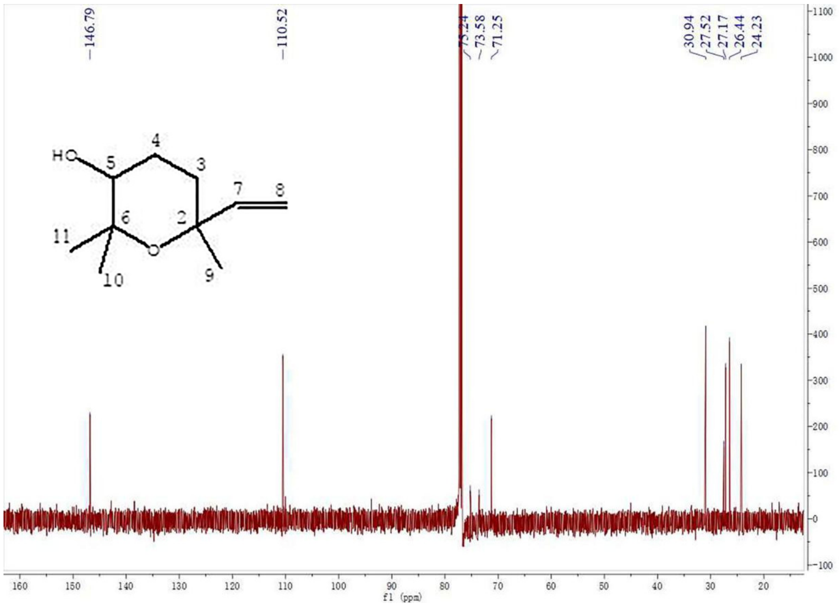

Figure S10. ^{13}C and ^{1}H NMR spectra for cryptone (21).

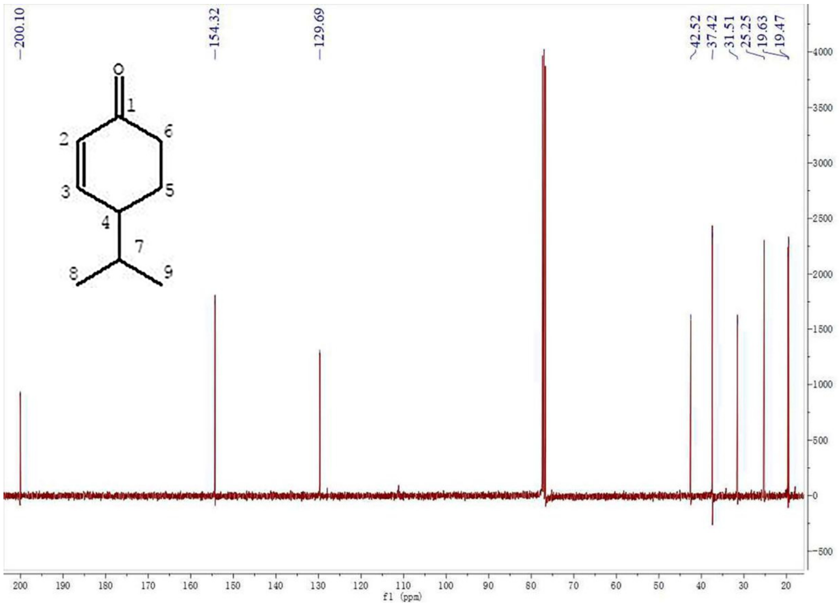

Figure S11. ^{13}C spectra for linalyl acetate (27).

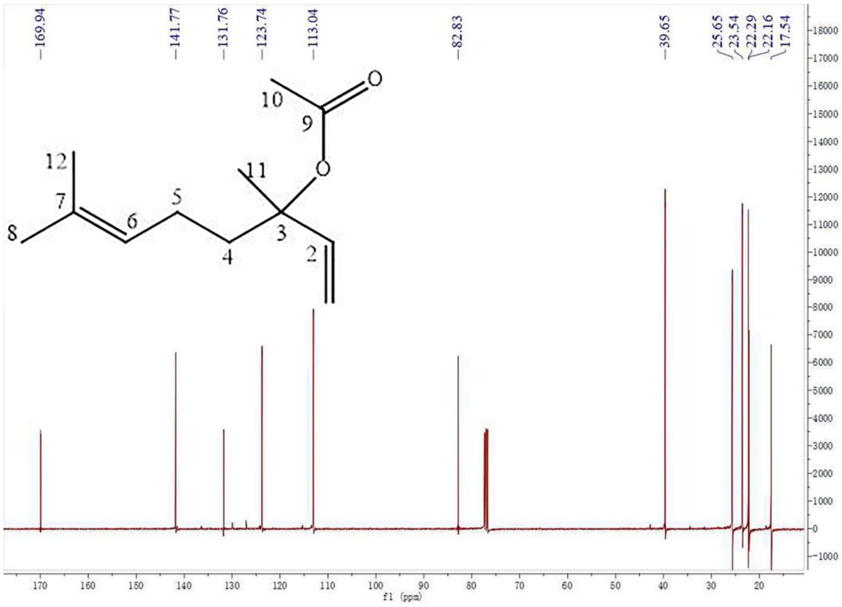

Figure S12. ^{13}C spectra for 3,7-dimethylocta-1,7-diene-3,6-diol (29).

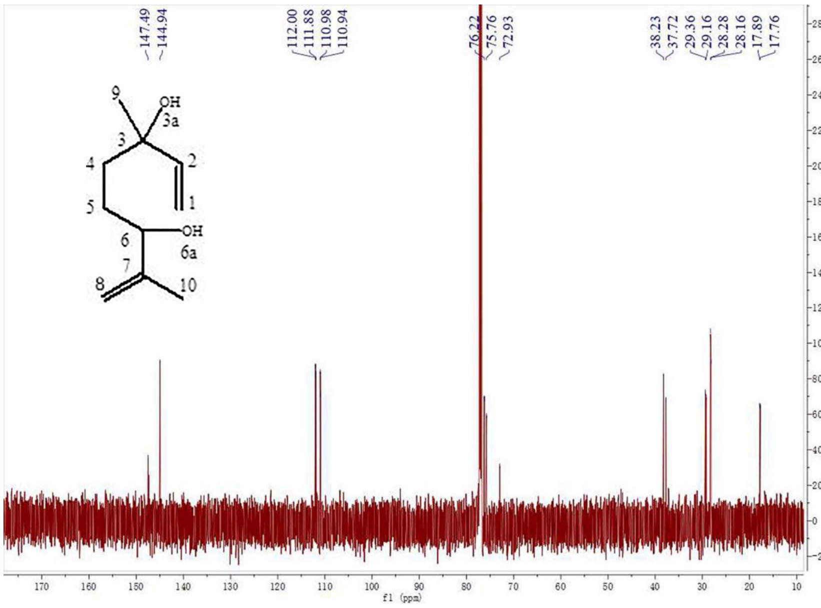

Figure S13. ^{13}C spectra for Lavandulol acetate (32).

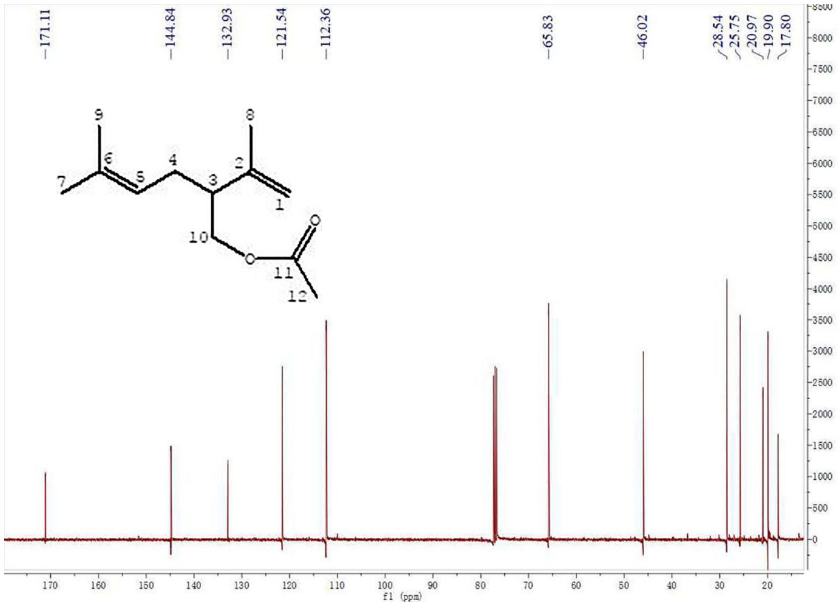

Figure S14. ^{13}C spectra for (E)-7-hydroxy-3,7-dimethylocta-1,5-dien-3-ylacetate (33).

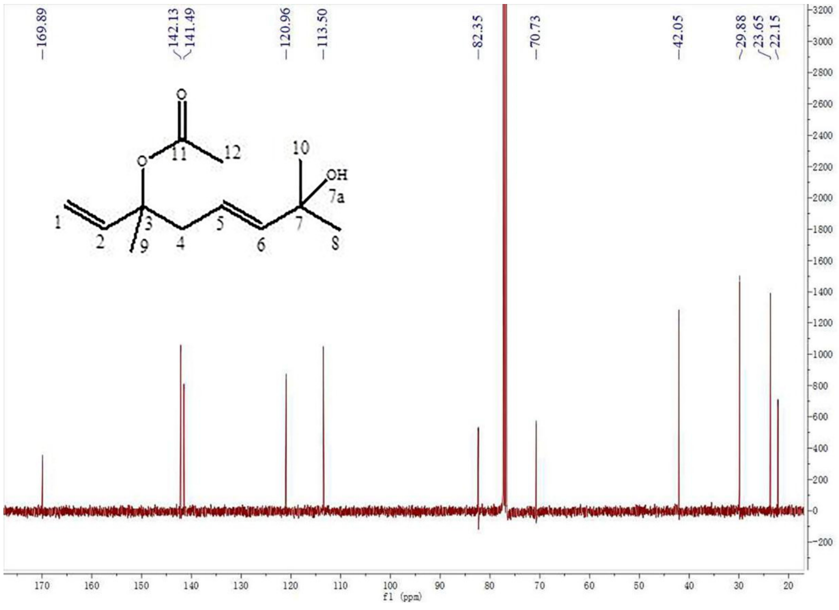

Figure S15. ^{13}C spectra for (E)-*β*-farnesene (38).

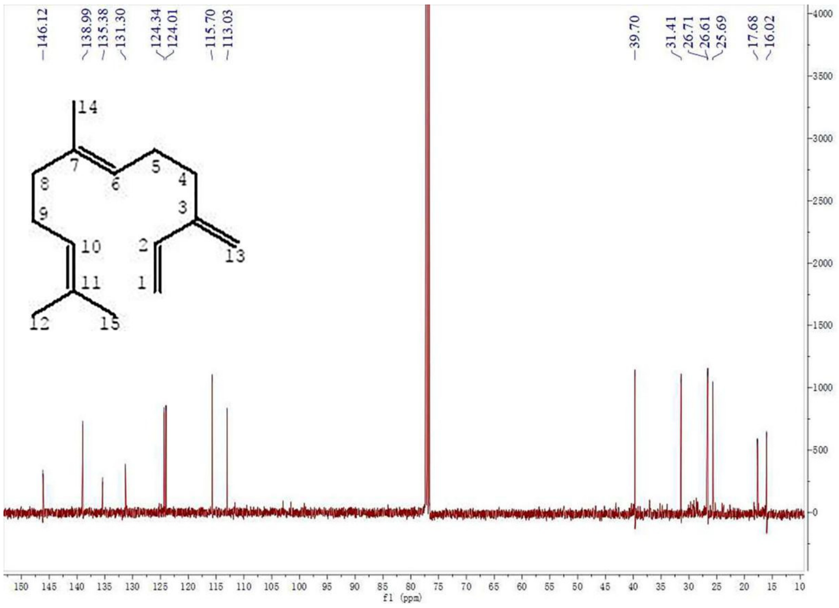

Figure S16. ^{13}C spectra for germacrene D (39).

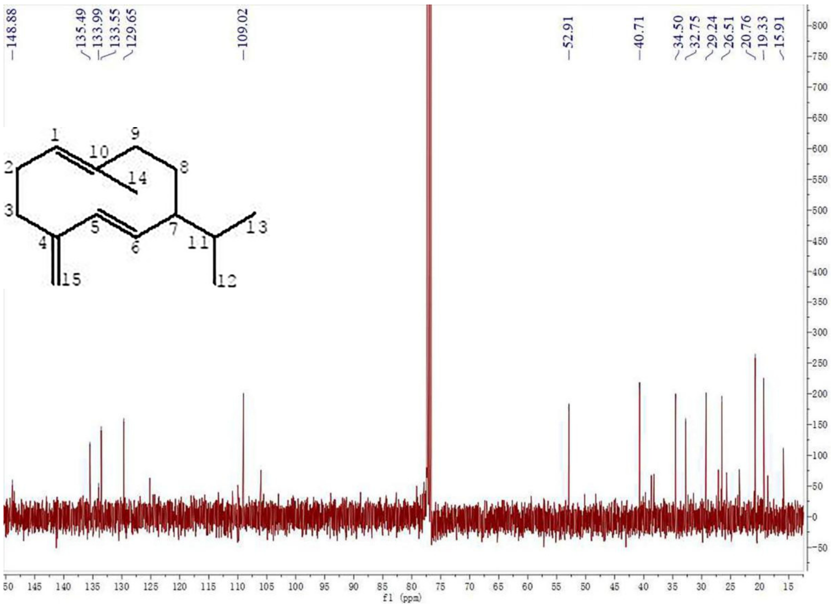

Figure S17. ^{13}C spectra for γ-cadinene (40).

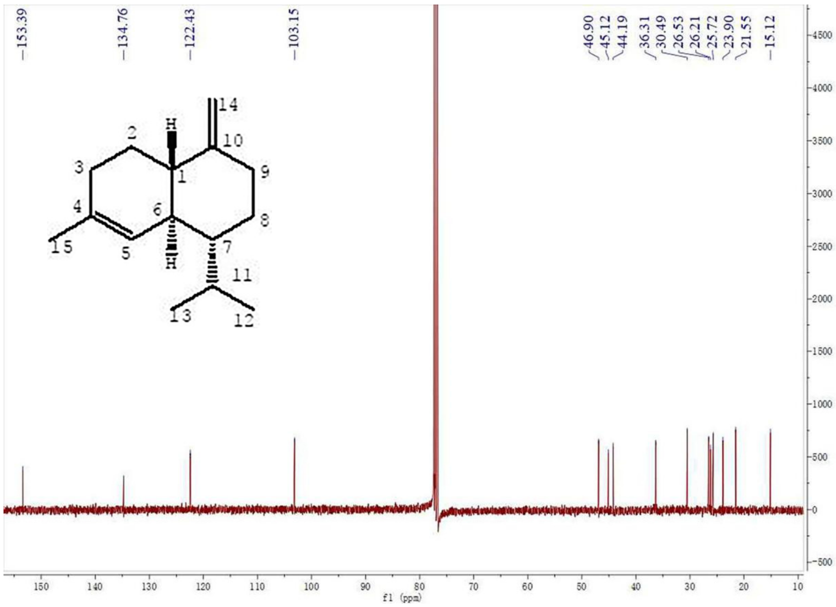

Figure S18. ^{13}**C spectra for 11-hydroxy-α-santal-9-ene (41).**

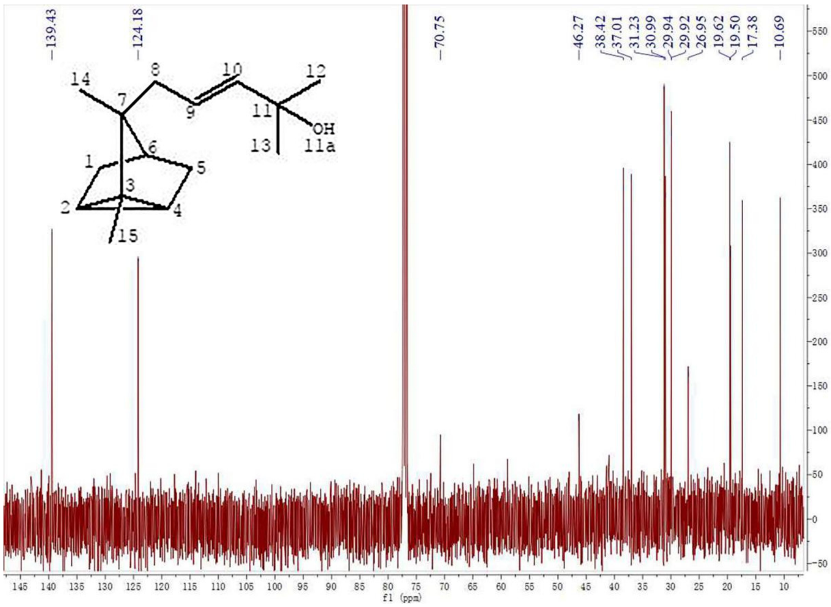

Figure S19. ^{13}C spectra for *β*-caryophyllene oxide (43).

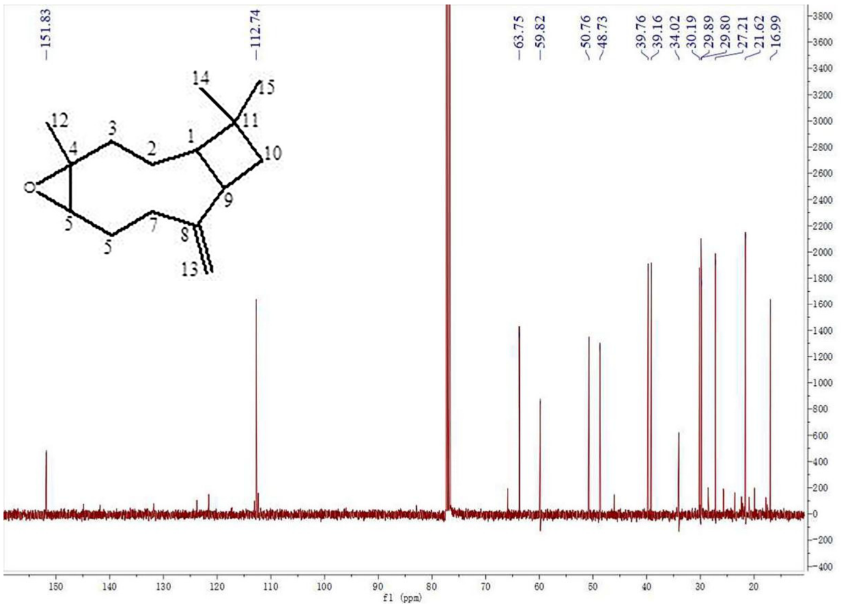

Figure S20. ^{13}C spectra for (3S,6R,9R)-2-(hydroxymethyl)-5,5,9-trimethyltricyclo[7.2.0.0 (3,6)]undecan-2-ol (45).

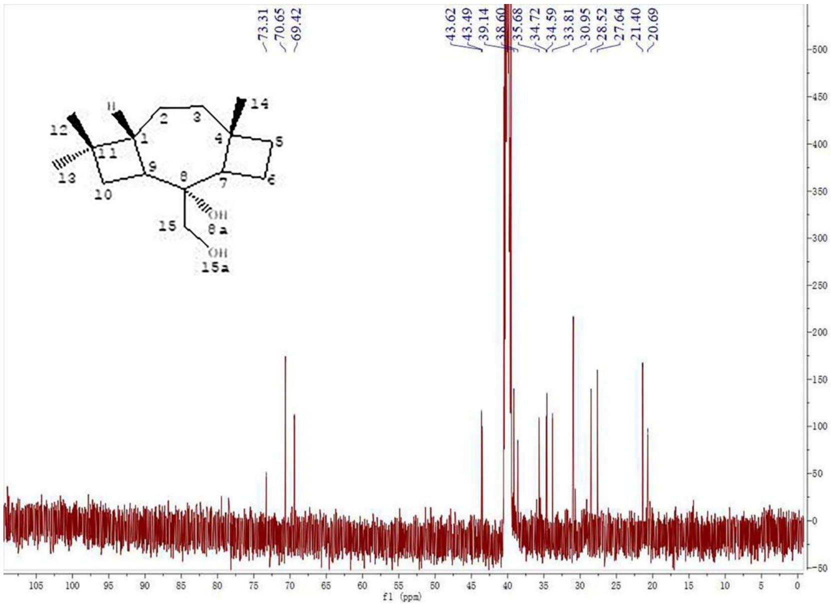

Figure S21. ^{13}C spectra for (6R,10R)-6,10,14-Trimethyl-2-pentadecanone (46).

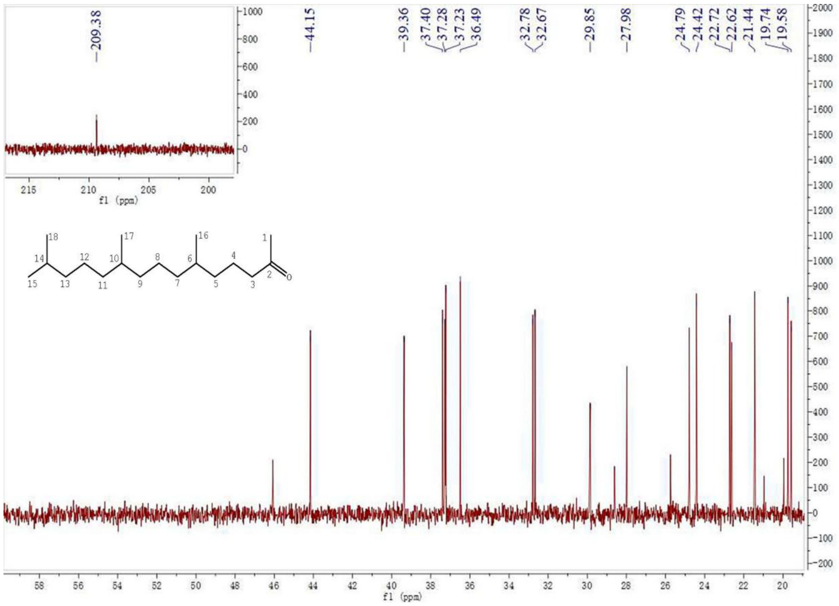

Supplement: Supplementary file 1 [file molecules-25-03166-s001.pdf]
